# “Mother and baby plate”: a strategy to improve stability in proximal fractures of the ulna

**DOI:** 10.1007/s00402-023-04979-8

**Published:** 2023-07-17

**Authors:** Stefanie Hoelscher-Doht, Nicola Zufall, Maximilian Heilig, Philipp Heilig, Mila Marie Paul, Rainer Heribert Meffert

**Affiliations:** Department of Trauma, Hand, Plastic and Reconstructive Surgery, University Hospital Würzburg, Oberdürrbacher Staße 6, 97080 Würzburg, Germany

**Keywords:** Ulna, Fracture, Monteggia, Plate, Double-plate, Mother–baby, Biomechanical

## Abstract

**Introduction:**

Proximal ulna fractures with a large zone of comminution, such as in the context of Monteggia injuries, require mechanically strong osteosyntheses as they occur in regions with high physiological joint load. Consequently, implant failure and pseudarthrosis are critical and devastating complications, especially with the background of mainly young patients being affected. An effective solution could be provided by adding a small second plate 90° angulated to the standard dorsal plate in the area of non-union. Thus, this study investigates whether, from a biomechanical point of view, the use of such a mini or baby plate is worthwhile.

**Materials and methods:**

Comminuted fractures distal to the coronoid process, equivalent to Jupiter type IIb fractures, are generated on artificial Sawbones^®^ of the ulna and stabilized using two different plate osteosyntheses: in the first group, a dorsal locking compression olecranon plate is used (LCP group). In the second group, a small, ulnar 5-hole olecranon plate is added as a baby plate in addition to the mother plate at the level of the fracture zone (MBP group). Dynamic biomechanical loading in degrees of flexion from 0° to 90° is carried out to determine yield load, stiffness, displacement, and changes in fracture gap width as well as bending of the dorsal plate.

**Results:**

The “mother-baby-plate” osteosynthesis had a significantly higher yield load (*p* < 0.01) and stiffness (*p* = 0.01) than the LCP group. This correlates with the increased movement of the proximal fracture element during cyclic testing for the LCP group compared to the MBP group as measured by an optical metrology system.

**Conclusions:**

Here, we show evidence that the addition of a small plate to the standard plate is highly effective in increasing the biomechanical stability in severe fractures equivalent to Jupiter type IIb. As it hopefully minimizes complications like pseudarthrosis and implant failure and as the additional preparatory effort leading to compromised blood supply is regarded to be negligible, this justifies and highly advises the use of a mother–baby-plate system.

## Introduction

**Fig. 1:**
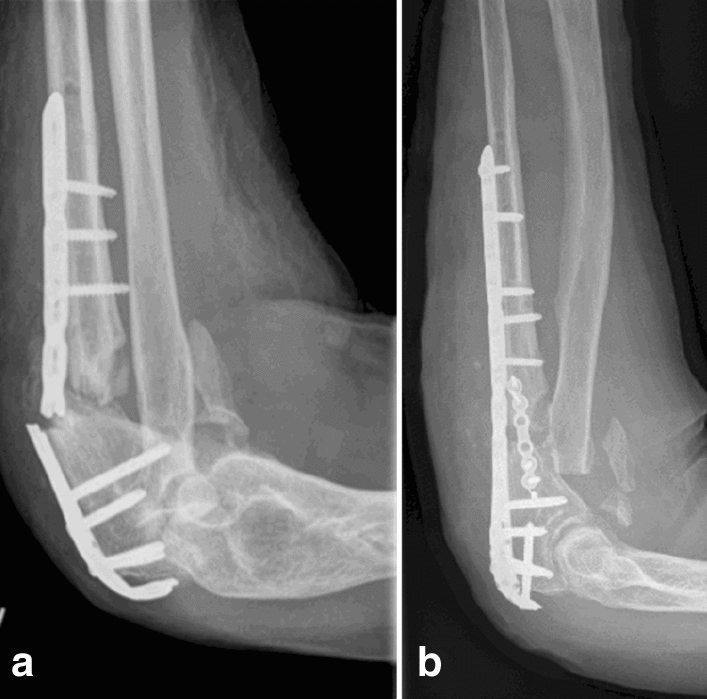
Complications like implant failure of the dorsal angle-stable plates (**A**) are seen in comminuted proximal ulna fractures. Here the case of non-union and subsequent plate breakage is presented. As a rescue operation, a mother–baby-plate osteosynthesis was performed (**B**)

Comminuted fractures and fracture fixation in poor bone quality confront the surgeon with major challenges. Particularly in regions close to joints with high physiological loading of the bone, such as distal femur fractures or proximal tibial fractures, comminuted fractures are often stabilized from more than one side. Additional plate osteosynthesis on the opposite side is regularly resorted to for tibial plateau fractures, and for distal femoral fractures depending on the fracture morphology. In addition to the lower extremity, comminuted fractures of the upper extremity also place high demands on osteosynthesis: for example, remarkably high forces act on the elbow joint even during movements in everyday life without loading or weight-bearing of the arm. During flexion movements alone without resistance, forces act on the joint that correspond to one to two times the weight of the forearm [1]. For example, simple activities, such as eating or clothing, already pose a load of approximately 300 N on the elbow joint. When supporting oneself, e. g. standing up from a chair, the forces acting on the elbow joint already reach 1700 N in the medial and 2500 N in the lateral joint region [2, 3]. The force picked up by the hand increases by a factor of 20 due to the small lever arms and the leverage effect of the arm in the elbow joint [2]. Considering these biomechanical principles of physiological forces at the elbow joint, it is not surprising that despite very stable plate osteosynthesis by a rigid dorsal plate, complex fractures of the proximal ulna still result in implant failure with consecutive plate breakage (Fig. 1A).

Especially in situations of surgical treatment of complex fractures, an additive second plate osteosynthesis should also be discussed at the proximal ulna. In proximal humerus fractures, double plate osteosynthesis is nowadays an established option for decisively increasing the stability of the osteosynthesis in difficult fracture conditions, especially in the absence of medial bony support, and thus counteracting complications [4–6]. There is a recurring debate as to whether double plate placement significantly reduces blood flow to the bone [7]. Whereas double plate osteosynthesis is already an established alternative to dorsal plate osteosynthesis in olecranon fractures [8–11], a dorsal plate is still the surgical standard for more distal ulnar fractures, like Monteggia injuries [12, 13]. It has not yet been investigated whether, a plate in addition to a dorsal plate offers a significant biomechanical advantage as shown in the rescue operation (Fig. 1B), as it does in other fractures. Therefore, basic biomechanical data are necessary to determine whether there is any significant difference from a biomechanical perspective in placing a second plate (baby plate) in addition to the dorsal plate (mother plate) in comminuted ulnar fractures. Fundamental biomechanical facts could extend the clinical use of double plate osteosynthesis beyond that in revision surgery and already become the new routine in primary care of comminuted fractures of the proximal ulna.

Therefore, the aim of this study was to biomechanically evaluate a mother–baby-plate system in comparison to single dorsal plating. Our hypothesis was that a double-plate osteosynthesis consisting of a dorsal locking compression plate (mother plate) and a single small lateral low-profile olecranon plate (baby plate) would have a higher biomechanical stability than a single dorsal locking compression plate alone. The mother–baby-plate method is intended to achieve greater stability without adding soft tissue irritation due to an additional large implant or increasing the area of bone preparation required intraoperatively.

## Materials and methods

### Specimens and fracture generation

A proximal ulna fracture with a 1 cm zone of comminution, equivalent to Jupiter type IIb fractures, was simulated in synthetic ulnae (4th Gen., Composite, No. 3426, Sawbones Europa, Malmö, Sweden), following previous work from literature [14–16]. The bones were fixed in 3D printed, experimental devices specially adapted to the bone and the plates used later. First, the simulation of the triceps tendon was attached to the olecranon [9, 17]: using a 3D-printed template for standardization, two 2.8 mm holes were drilled in at the olecranon tip and then a 2.0 mm stainless steel wire (Hamburger Tauwerke Fabrik GmbH & Co.KG, Germany) was inserted in a U-shape. To prevent cut-through of the wire during biomechanical testing, washers were used as described in preliminary work [9]. Then, the plates were attached. The holes were drilled using a template and the screws were placed in a standardized manner. For fracture generation, an oscillating saw was used.

### Experimental groups and fixation methods

**Fig. 2:**
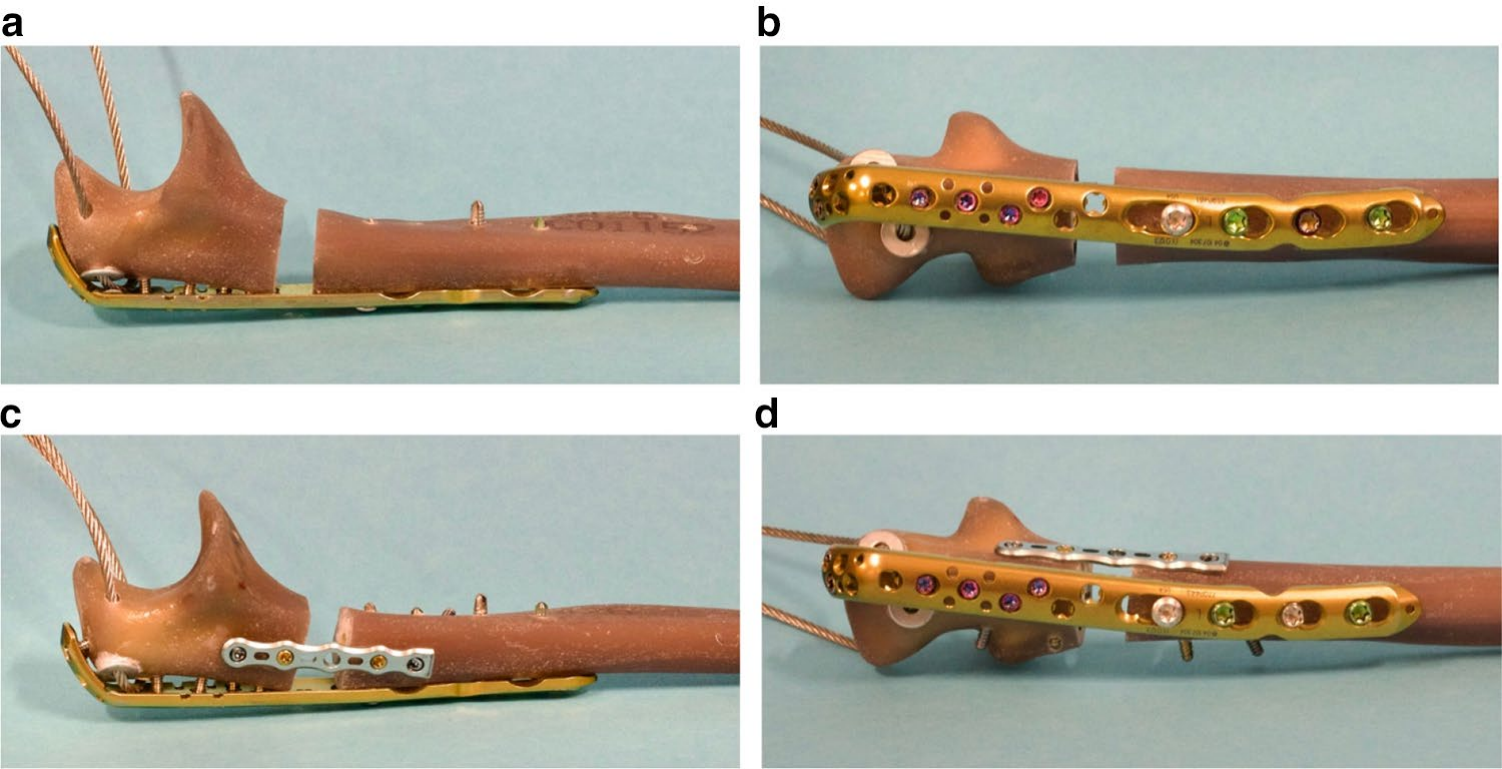
In group 1, the comminuted ulna fracture was stabilized with a standard dorsal plate osteosynthesis (**a** + **b**). In group 2, additionally a baby-plate (5-hole ulna plate) was attached at the ulnar side with 4 screws (**c** + **d**)

Two different plate osteosyntheses were selected to stabilize comminuted fractures of the proximal ulna: in group 1, a dorsal plate commonly used in clinical practice was applied: the VA-LCP olecranon plate 2.7/3.5 L116 mm (DePuy Synthes, Johnson & Johnson Medical GmbH, Norderstedt, Germany). A total of six screws were placed proximal to the fracture and four screws distal to the fracture (Fig. 2a + b). In group 2, a TriLock olecranon 7-hole plate 2.8 shortened to five screw holes (baby plate) was placed from the ulnar side in addition to the dorsal plate used in group 1 (mother plate) (Fig. 2c + d). The two screw holes far from the fracture were loaded with angle-stable screws, and those two, close to the fracture were loaded with non-angle-stable screws (Fig. 2c).

In accordance with the Institute of Epidemiology and Biometry at the University of Würzburg, a priori power analysis was performed. With the specification of a statistically significant difference at a significance level of 5% and a power of 80% in the independent *t* test, a total number of 24 artificial bones was determined, resulting in a respective group size of 12.

### Biomechanical test set-up

The primary stability of the two different osteosyntheses was investigated in dynamic loading tests: based on a pre-test series on bones with ulnar defect fractures and stabilization via a dorsal plate osteosynthesis, the definitive parameters such as the force levels and the respective number of cycles of the biomechanical testing were determined (Table 1). The sequence of the biomechanical testing with loads in different degrees of flexion was based on a previous work of our research group [9]. The main focus of our investigations was the detection of the transition from elastic to plastic deformation (yield load) on the basis of increasing loads in 90° flexion position.

**Table 1:** The exact test modalities for the biomechanical test set-up are demonstrated

Degree of flexion	Number of cycles	Force (N)
0°	10 settling cycles	5–20
0°	500	10–150
30°	300	10–150
60°	300	20–300
90°	Until failure	300 + 5 N/cycle

**Fig. 3:**
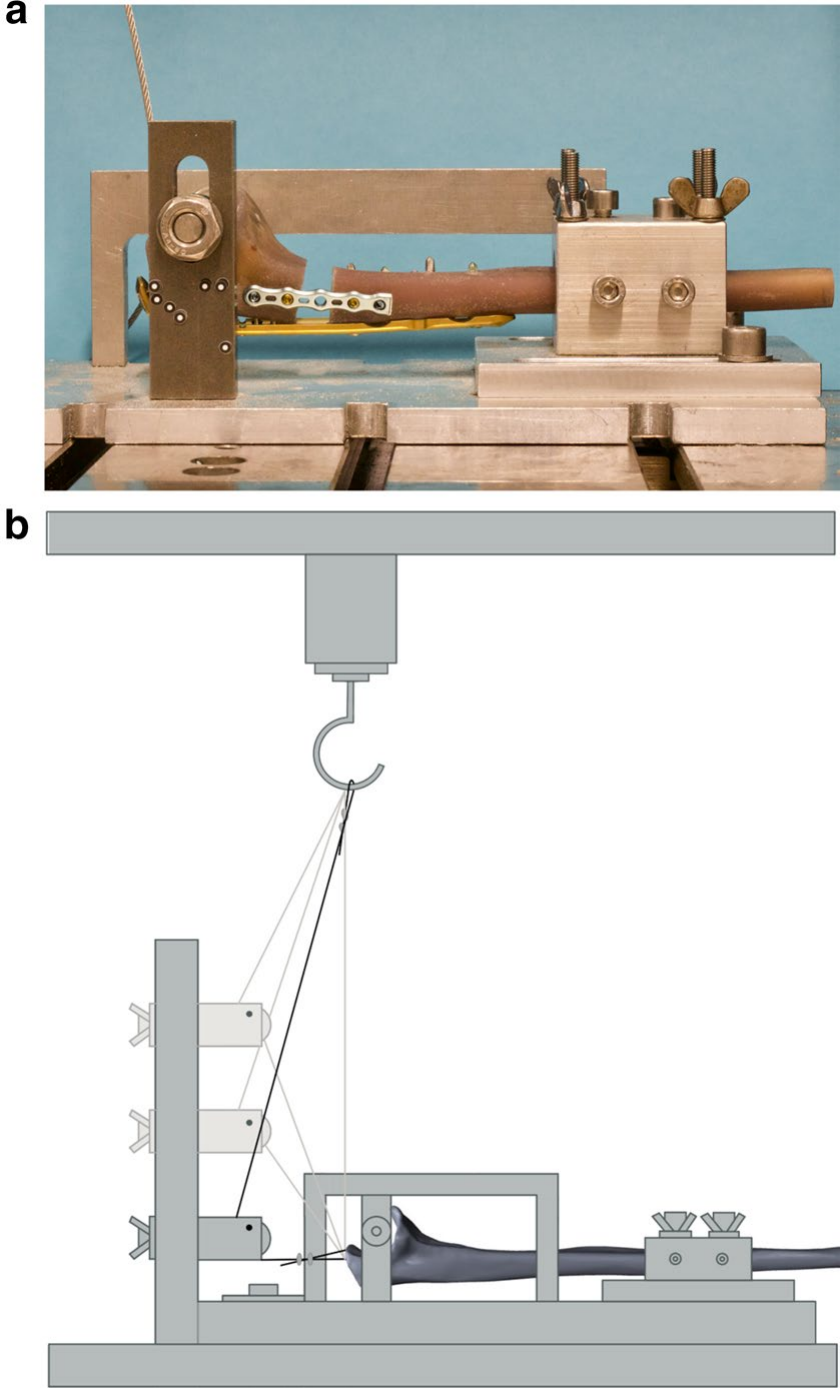
The biomechanical tension loading was performed in 0°, 30°, 60° and 90° flexion of the elbow by a deflection roll

After 10 setting cycles with a force interval of 5–20 N at a speed of 5 mm/min, cyclic testing was performed at 0°, 30°, 60° and finally at 90° flexion (Fig. 3) of the elbow joint. The biomechanical test set-up is well in agreement with previous studies in literature [15, 17–20].

### Optical system

To analyze the exact location of movement, a three-dimensional camera system (ARAMIS 3D Professional Carl Zeiss GOM Metrology GmbH, Braunschweig, Germany) was used to capture a photo of each cycle at the time of maximum force application at 90° flexion. The distance between optical marker points was calculated between the first test cycle and the test cycle of the respectively determined yield load at 90°, i.e., until plastic deformation was detected.

### Parameters of interest

The displacement during cyclic testing was tracked by the traverse of the materials testing machine. Yield load represents the force at the associated point on a force-strain curve, which indicates the end of elastic behavior and the onset of plastic behavior. The yield load was determined during testing at 90° flexion. This point is highly relevant for a possible implant failure, since from this point on the deformation can no longer adapt to its initial state. The stiffness corresponds to the slope of the graph during elastic deformation and is calculated by two points in the force–displacement diagram.

Selected parameters of interest determined by the optical system are the fracture gap width at the proximal and at the distal part of the osteotomy gap, which are defined as D1 and D3 (Fig. 5a). Additionally, the displacement of the proximal fracture element based on points P1, P2 and P3 which refer to the relative movement to the distal part of the bone. Furthermore, displacement and bending of the dorsal LCP plate at points P8, P9 and P10.

### Statistical analysis

Statistical analysis was carried out in collaboration with the Department of Clinical Epidemiology and Biometry at the University of Würzburg and calculated using SPSS^®^ software V.28 (IBM^®^, NY, USA). To determine a significant difference between the findings of the test parameters, a one-sided independent *t* test was performed. In the event in which the normal distribution assumption was not met, the Wilcoxon–Mann–Whitney test was performed. Statistical significance was set at *p* < 0.05.

## Results

**Fig. 4:**
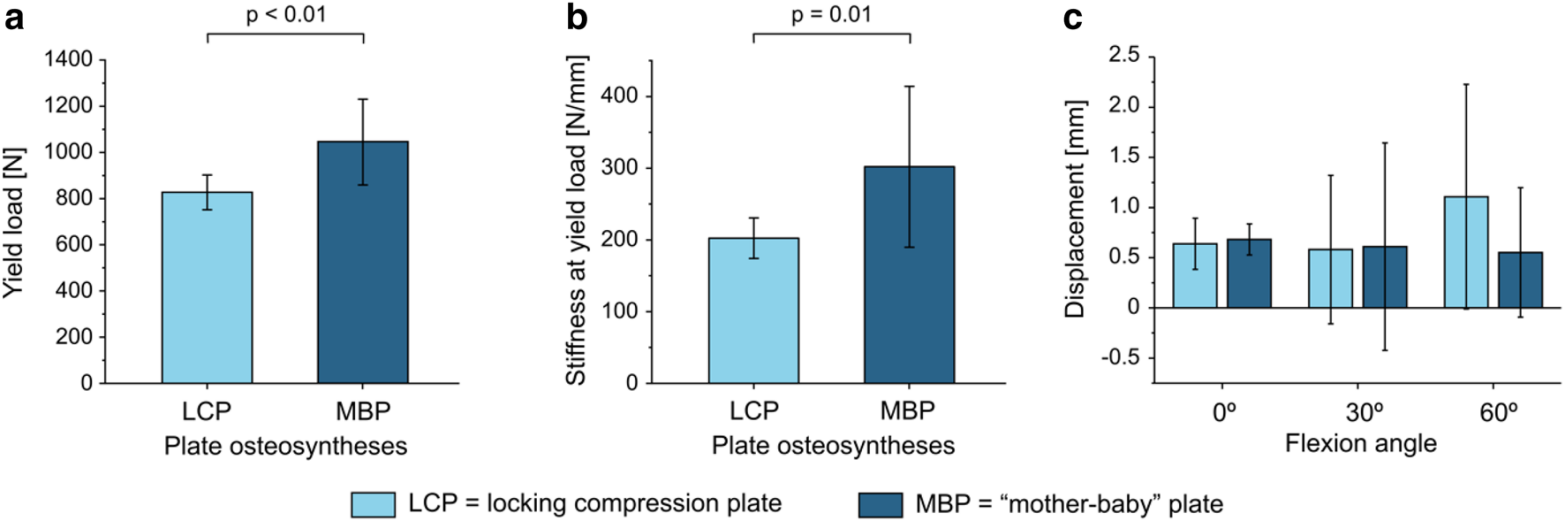
Biomechanics of plate osteosyntheses of the two groups LCP and MBP indicate a significant difference in yield load (**a**, *p* < 0.01) and stiffness at yield load (**b**, *p* = 0.01). **c** Biomechanical testing of the displacement of LCP versus MBP in different flexion angles. No significant difference in displacement between LCP and MBP was observed

Out of a total of 12 bones tested for each group the mean yield load for group 1 (LCP) was 827.11 N (SD: 74.93 N) and for group 2 (MBP) 1045.5 N (SD: 186.04 N) (Fig. 4a), presenting a statistically significant difference (*p* < 0.01). The corresponding mean values for stiffness at yield load are 202.41 N/mm (SD: 28.23 N/mm) for group LCP and 302.12 N/mm (SD: 112.27 N/mm) for group MBP (Fig. 4b). The groups were found to be statistically significantly different (*p* = 0.01). Both yield load and stiffness at yield load are significantly higher for the mother baby plate system withstanding higher forces without plastic deformation and providing higher stability.

No significant difference was found for the displacement, recorded by the traverse of the material testing machine, of the flexion angles of 0°, 30° and 60° (Fig. 4c).

### Results from the optical system

**Fig. 5:**
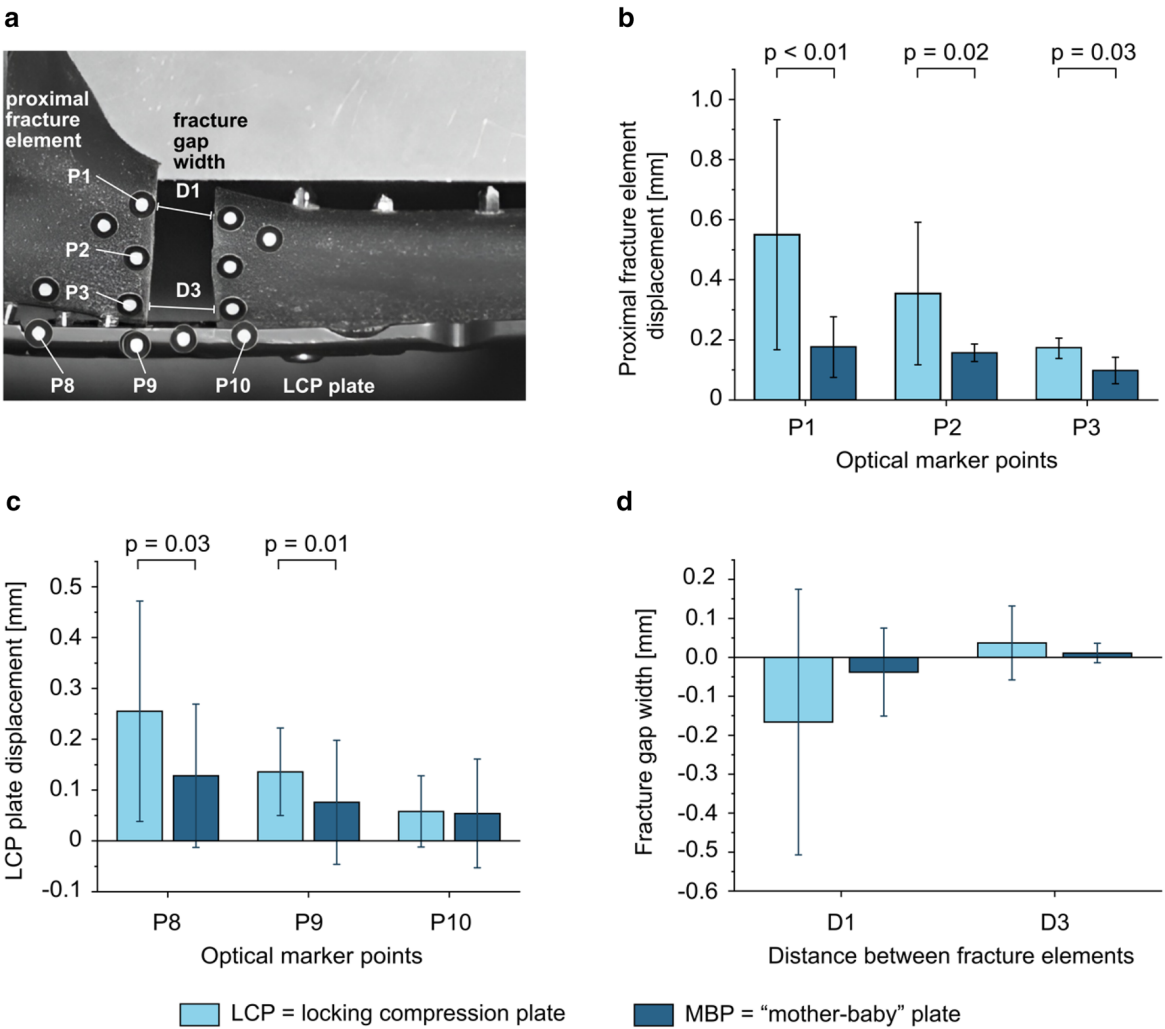
**a** Overview of marker points analyzed by the optical system. **b** A significant higher displacement of the analyzed points on the proximal fracture element could be detected for the LCP plate alone in comparison to the mother–baby-plate group (MBP) for P1 (*p* < 0.01), P2 (*p* = 0.02) and P3 (*p* = 0.03). **c** The displacement of points on the LCP plate is significantly higher for the LCP group compared to the MBP group for P8 (*p* = 0.03) and P9 (*p* = 0.01). There is no significant difference for P10. **d** Compared to the MBP group, the LCP group shows a higher movement of both fracture elements, as the proximal fracture gap distance D1 decreases and the distal fracture gap distance D3 increases, although no significant difference could be found

The displacement of the proximal fracture element is significantly higher in the LCP group at all points P1, P2 and P3. It can also be seen that the movement is most pronounced at P1 with a statistically difference in-between groups of *p* < 0.01, followed by P2 (*p* = 0.02) and finally P3 (*p* = 0.03) (Fig. 5b). Thus, the greatest motion occurs in the ventral portion of the fracture area in which no stabilization is given. In the MBP group, the movement of this fracture element is much smaller.

The movement of the optical marker points P8 and P9 located on the LCP plate have shown a significantly lower displacement for the MBP group [P8: (*p* = 0.03); P9: (*p* = 0.01)] (Fig. 5c). The optical marker point P10 on the LCP plate showed no significant difference in displacement values for each group. The movement of these points represent the deformation of the LCP plate. P8 and P9 are closest to the point of force application through the simulation of the triceps tendon and are thus subjected to large movement. In the MBP group, it can be seen that the additional small plate is able to effectively absorb the deformation and force application at the LCP plate resulting in lower displacement of the optical marker points.

The fracture gap width showed a greater amount of movement in the LCP group. However, this did not present a statistically significant difference to the MBP group (Fig. 5d). In general, it can be seen that the width of the proximal fracture gap decreases at distance D1, while the values of the fracture gap movement of D3 lie in the positive range. This shows that the fracture elements approach each other due to tilting of the proximal fracture element.

## Discussion

The elbow joint is the second most frequently luxated joint in adults. Fractures of the proximal ulna in combination with proximal radioulnar dissociation and dislocation of the radial head are referred to as Monteggia fractures. Monteggia injuries occur with an incidence of 2–5% of all forearm injuries [21]. Among adults, Monteggia fractures most commonly arise in young males due to high-energy traumata, such as falls from great heights, sports injuries, or car accidents, whereas fractures in elderly females tend to be caused by low-energy traumata [22]. The bones used in this study correspond to those of healthy young people, i.e., one of the two age groups in which these injuries occur most frequently. The use of synthetic bones ensured a high degree of standardization and a lack of variability between the specimens and is an established method for biomechanical testing in vitro [9, 14, 16]. Therefore, the significance of the results and the comparability of the groups is considerably higher than when human specimens are used. Standardization was additionally increased by using customized 3D-printed experimental jigs for plate placement and screw fixation.

Despite improved biomechanical understanding and advances in surgical fracture care, complications, revision surgery and unsatisfactory functional outcomes after Monteggia fractures are still common and can cause significant damage with loss of function if inadequately treated [23]. Main treatment principles are open reduction and anatomic reconstruction of the ulnar fracture, followed by reduction of the radial head dislocation. This is followed by treatment of associated injuries (e.g., radial head fractures, coronoid fractures, LCL ruptures). Dorsal plate osteosynthesis has been a popular surgical procedure for the treatment of intra- and extra-articular multiple fragment fractures of the olecranon for a considerable number of years. These are positioned on the dorsal face of the ulna to withstand the flexion forces imposed by the triceps tendon. Locking compression olecranon plates (LCP) from DePuy Synthes (Johnson & Johnson, USA) like used in this study are a typical example for a commonly used dorsal plate type [24]. Most companies offer dorsal plates for proximal ulna fractures. General complications that often lead to revisions are a high risk of wound healing problems, chronic pain symptoms, limitation of movement, irritation of the surrounding soft tissue [23]. Despite stable osteosynthesis, nearly one-third of cases of revision surgery after Monteggia injuries are performed because of pseudarthrosis of the proximal ulna [23]. Therefore, we deliberately chose a fracture simulation with comminuted zone and an implant of an angular stable dorsal plate osteosynthesis frequently used in the clinical practice for our test setup to represent these critical situations with bony defect zone and strong loads on the osteosynthesis in-vitro. Both fracture simulation and simulation of triceps tendon forces are established methods from previous studies that we have tested in the laboratory in our pre-test series [9, 16]. Tear out of the wire strapping used to simulate the triceps tendon was not seen in our testing. The simulated forces, primarily of traction through the triceps tendon, as described in previous biomechanical studies, are the relevant forces for an in vitro model to focus on [9, 14, 25–27].

In the load-to-failure tests at 90°, however, there was no plate breakage but rather pronounced bending of the plate with fracturing of the bone around the screws and, in some cases, additional avulsion of the simulated triceps tendon. This was not useful for demonstrating cyclic failure of the implant as seen clinically (see Fig. 1) and a modification of the test set-up at 90° was necessary. Biomechanically relevant is the region at which the elastic deformation changes to plastic deformation. Therefore, the test protocol was adjusted accordingly with increasing forces and the point of yield load, the change from elastic to plastic deformation, was determined.

For this most fundamental parameter of our investigations, a significantly higher yield load could be observed for the mother–baby-plate compared to dorsal plate osteosynthesis alone. This means that even with the small mini-implant selected here, in addition to the dorsal plate, the clinically highly relevant point of onset of plastic, irreversible deformation was significantly increased by an average of more than 200 N. Corresponding to this, the evaluation of the optical system revealed reduced movement of the fracture gap and proximal fracture fragment as well as reduced bending of the dorsal plate osteosynthesis for the combination with the miniplate. The results are thus conclusive in showing the strong biomechanical effect of the mother–baby-plate system, which implies less complications as implant failure and pseudarthrosis in clinical practice. Especially in the ventral fracture region, the baby plate provides sufficient stabilization Besides the dorsal plate osteosynthesis, new low-profile plates that are inserted on both sides laterally and medially on the olecranon are increasingly becoming a popular alternative in proximal ulna fractures, to avoid soft tissue irritation [9, 10, 24, 28, 29]. In clinical practice, these plates provide advantages such as a reduction in soft tissue irritation with subsequently less wound healing problems. In our clinical experience, a more extensive dissection of the ulnar nerve will not normally be necessary in double plating, as well as transposition of the nerve. General advantages of double plate osteosynthesis include a better distribution of forces, increased primary stability and additional options of fixation possibilities. However, the general principle of double plate osteosynthesis is also associated with disadvantages: an expansion of the exposed preparation area may be required leading to additional preparation of the bone and resulting in a prolonged operation time and potentially higher complication rate, due to increased amount of osteosynthesis material. In past studies investigating olecranon fractures, double plate osteosynthesis and dorsal plate osteosynthesis of the ulna have often been shown to have similar biomechanical stability [9]. Nevertheless, a dorsal implant often has the clinical advantage of covering a larger bone area due to its additional length on the ulnar shaft and thus often represents the better alternative to the surgeon. In addition, the screw locations of the dorsal plating are favorably situated due to the proximal extension of the plate around the olecranon and several options that aim exactly perpendicularly into the coronoid process, thus providing excellent stabilization.

In addition, the small plate applied might help to maintain anatomical reduction for the later bridging plate. The plates chosen in this study are approved and established implants for the elbow with sufficient plate thickness and screw size. A miniplate with four screw options was chosen after clinical evaluation of different implant lengths and sizes. As known from all regions of fracture treatment, there must be a well-balanced approach between stability by osteosynthesis and nevertheless biological maintenance for the complete healing of a fracture [30]. Therefore, the use of two plates at the proximal ulna must also be critically discussed under biological aspects of compromising the blood supply. However, from our clinical practice and surgical experience, we consider the additional risk of compromising the blood supply to be extremely low when using the small additional plate presented in this study:

The additional bone exposure at the level of the fracture is significantly reduced due to the shortening of the plate, respectively, the exposure of the bone at the level of the fracture is already present during the initial preparation without the need for any additional trauma at all. The screws of the baby plate close to the fracture are deliberately set non-angularly stable to theoretically allow oscillation in the fracture area and not to set the mini-implant too rigidly. This screw combination and selection already showed significantly higher stiffness for the MBP group in addition to increased yield load and reduced fracture motion. Theoretically, the biomechanical effect of higher stability would even be increased if the baby plate was filled with four angle-stable screws.

These biomechanical data demonstrate that the use of an additional baby plate in comminuted fractures of the proximal ulna is crucial to prevent early irreversible changes in osteosynthesis and thus increase the chances of healing and reduce implant failure. In cases where there is a large, comminuted zone of the proximal ulna or in cases of revision surgery, mother-baby plate osteosynthesis is an alternative to singular dorsal plate osteosynthesis, that provides high stability. However, we see it as a benefit of options for the surgeon in special cases rather than a new standard of fracture care for Monteggia injuries.

### Limitations

This study is a biomechanical in-vitro study design and only focused on primary stability. The study did not include the effects of fracture healing or soft tissue irritation. By using a biomechanical test model, the conditions in vivo are reproduced as accurately as possible. However, the clinically occurring anatomical variations cannot be replicated. Another limitation is the use of synthetic bones, which correspond to the biomechanical properties of healthy bone. High reproducibility and very accurate experimental performance (via 3-D printed devices for implant anchorage, etc.) could be achieved by using them. However, synthetic bones can only ever represent one aspect and further verification on human bone should follow in the long term.

## Conclusions

Overall, the results show that the mother–baby-plate system is biomechanically more stable and can withstand greater forces than the sole dorsal LCP plate. Especially the findings using the optical metrology system suggest that the MBP group is significantly effective in reducing fracture gap movement and thus knocks off complications such as pseudarthrosis and implant loosening. As for the used small baby plate with only four screws, no large additional tissue preparation is required and the effect on the blood supply is considered negligible, this study highly advocates the use of a mother–baby-plate system for the benefit of patients with severe proximal ulna fractures.

## Data Availability

Data can be made available upon request from the corresponding author.
